# Potassium channel openers and prostacyclin play a crucial role in mediating the vasorelaxant activity of *Gynura procumbens*

**DOI:** 10.1186/1472-6882-13-188

**Published:** 2013-07-23

**Authors:** Hien-Kun Ng, Ting-Fung Poh, Sau-Kuen Lam, See-Ziau Hoe

**Affiliations:** 1 Department of Physiology, Faculty of Medicine, University of Malaya, Kuala Lumpur, Malaysia

**Keywords:** *Gynura procumbens*, Vasodilation, Calcium channel, Potassium channel, Prostacylin

## Abstract

**Background:**

Previous studies of *Gynura procumbens* (*G. procumbens*) have shown that partially purified fractions of the leaves are capable of lowering the blood pressure of rats by inhibiting angiotensin-converting enzymic activity and causing vasodilatation. The objectives of this study were therefore to further purify the active compounds that exhibited selective effects on blood vessels, determine the mechanism of actions, and to qualitatively analyse the putative compounds present.

**Methods:**

The butanolic fraction (BU) of the crude ethanolic extract was purified using column chromatography to obtain several sub-fractions of different polarities. The *in vitro* effects of BU and the sub-fractions on vascular tension were subsequently determined using isolated rat thoracic aortic rings. The most potent sub-fraction (F1) alone was then investigated for its mechanisms of the vasorelaxant activity. In another experiment, thin-layer chromatography was used to qualitatively analyse the active compounds found in F1.

**Results:**

The BU and the sub-fractions ranging from 10^-7^ to 10^-2^ g/ml significantly (p < 0.05) inhibited the sustained tonic contractions induced by phenylephrine and potassium chloride in a concentration-dependent manner with various degree of potency. The most potent sub-fraction (F1) antagonised the calcium-induced vasocontractions (1 x 10^-4^ – 1 x 10^-2^ M) in calcium-free with high concentration of potassium as well as in calcium- and potassium-free Krebs-Henseleit solutions. Contractions induced by noradrenaline and caffeine were not affected by F1. The vasorelaxing effect caused by F1 was significantly attenuated with preincubation of potassium channel blockers (glibenclamide and 4-aminopyridine) and prostacyclin inhibitor (indomethacin) while it was not affected by preincubation with tetraethylammonium, l-nitro-arginine methyl esther, propanolol, atropine, oxadiazolo quinoxalin one and methylene blue. The qualitative phytochemical analysis of F1 indicated the presence of flavonoids.

**Conclusion:**

These results confirm previous findings that *G. procumbens* causes vasodilatory effects by blocking calcium channels. In addition, the present study further demonstrates that the vasodilatory effect of *G. procumbens* may also be due to the opening of potassium channels and the stimulation of prostacyclin production. The putative compounds are probably flavonoids in nature.

## Background

Hypertension is defined as a condition where there is persisting high arterial pressure with systolic and diastolic blood pressure (BP) exceeding 140 mmHg and 90 mmHg respectively [1]. Hypertension is a high prevalent risk factor for cardiovascular diseases (CVD) throughout the industrial world and it is becoming a common health problem worldwide because of increasing longevity and the prevalence of contributing factors such as obesity, physical inactivity, and unhealthy diets. Cardiovascular disease is responsible for one third of global deaths and is a leading contributor to the global disease burden [2]. Therefore, the search for a safe and effective method for controlling hypertension has been a continuous effort world-wide. In addition to conventional synthetic drugs like thiazide and captopril [3], naturally occurring compounds from plant origin have also been highly sought by researchers.

One of the major determinants of BP is the total peripheral resistance (TPR), which is determined by the contractile state of blood vessel or vascular tone. In view of its defining role in regulating BP, vascular reactivity has become a target in treating hypertension. With the advent of the direct vasodilator, hydralazine, in the early 1950s [4], many drugs that can cause vascular relaxation have been developed, including some plant-derived compounds.

*Gynura procumbens (G. procumbens*) (Lour.) Merr. or the local names of *sambung nyawa* (Malay) or *jian wei feng* (Chinese), from the family of Compositae, and a fast growing herbaceous plant, is found in Borneo, Java, the Philippines and Peninsular Malaysia. The plant is widely used to treat kidney diseases, rashes and fever and the leaves of the plant have been used in folk medicine as an antihypertensive agent [5].

A lot of effort and studies have been carried out in order to scientifically prove the pharmacological properties of *G. procumbens*. Ethanolic extract of *G. procumbens* leaves have been shown to possess antihyperglycaemic and antihyperlipidaemic activities in diabetic rats [6,7], and the *n*-butanol fraction from the *G. procumbens* leaves also has hypoglycaemic effects [8]. Another study had shown that hexane and ethyl acetate fractions have potential in stimulating glucose uptake in 3 T3-F44 adipocytes [9]. Steroids isolated from the plant have been proven to possess anti-inflammatory activity [10]. Furthermore, ethanolic extract of it has demonstrated anti-ulcerogenic activity [11] and was able to inhibit ultraviolet (UV) B-induced matrix-metalloproteinase expression in human dermal fibroblasts [12]. Methanolic extract of *G. procumbens* has been categorised as a no-observed-adverse-effect level (NOAEL) crude drug that acts harmlessly and is considered to be of no toxicological concern [13]. Its aqueous extract appears to inhibit human mesangial cell proliferation [14].

*Gynura procumbens* has also been found to demonstrate antihypertensive activities in rat [15] by inhibiting ACE activity [16] and cause vasodilation via inhibition of calcium channels [17].

The main purpose of the present study was to further purify the active compounds that exhibit selective effects on the blood vessels, determine the mechanism of actions, and to qualitatively analyse the putative compounds present.

## Methods

### Plant material

Fresh *G. procumbens* were collected from the southern part of Peninsular Malaysia and authenticated by the Institute of Biological Sciences, University of Malaya. A voucher specimen (KLU 047690) had been deposited in the Herbarium at Rimba Ilmu, University of Malaya.

### Animals

Adult male SD rats, weighing from 200 – 300 g, were obtained from the Experimental Animal Center, University of Malaya. All the rats were kept under standard condition, given standard rat chow and tap water *ad libitum*. All experimental procedures that involve animal studies were approved by the University of Malaya Medical Center Animal Ethics Committee (FIS/14/10/2009/NHK (R)).

### Drug and chemicals

For extraction and fractionation processes, ethanol, ethyl acetate, hexane, methanol, and *n*-butanol were purchased from R & M Marketing, Essex, U.K. For the *in vitro* aortic ring pharmacological intervention, 4-aminopyridine, acetylcholine chloride (ACh), ACE, atropine sulphate, Brilliant Blue-G dye, caffeine anhydrous, glibenclamide, indomethacin, L-nitro-arginine methyl ester (L-NAME), noradrenaline (NA), oxadiazolo quinoxalin one (ODQ), phenylehprine (PE), propranolol hydrochloride, tetraethylammonium (TEA) were purchased from Sigma-Aldrich CO., St Loius, MO, USA. Ingredients for Krebs-Henseleit (K-H) solution were purchased from Merck KGaA, Darmstadt, Germany. All water-soluble drugs were dissolved in the distilled water before use while the water-insoluble drugs were dissolved in 15% tween 80 solution.

### Extraction and purification

The leaves of the plant were washed, cleaned of adulterants and dried in an oven at 40°C for 72 hours. The dried leaves were then ground into fined powder using a grinder. Crude ethanolic extract was obtained by macerating the powder with 95% ethanol at room temperature for 3 days. The extract was then concentrated by using rotary evaporator and reconstituted in 80% aqueous ethanol. The resulting fraction was then partitioned with hexane for the removal of the lipid or non-polar compounds. The hexanic phase was collected and dried *in vacuo* to produce the hexanic fraction (HX). The aqueous ethanolic phase obtained was subjected to evaporation to remove the ethanol and produce the aqueous solution that contained ethanol-soluble precipitate (EH). The precipitate was then filtered out and the remaining solution was partitioned again with water-saturated *n*-butanol. The butanolic phase was then dried using the rotary evaporator and lyophilised to obtain BU whilst the remaining aqueous phase was lyophilised to obtain the final aqueous fraction (FA). Since the fraction BU showed the highest vasorelaxing activities from previous studies [17], it was subjected to further purification.

**Figure 1:**
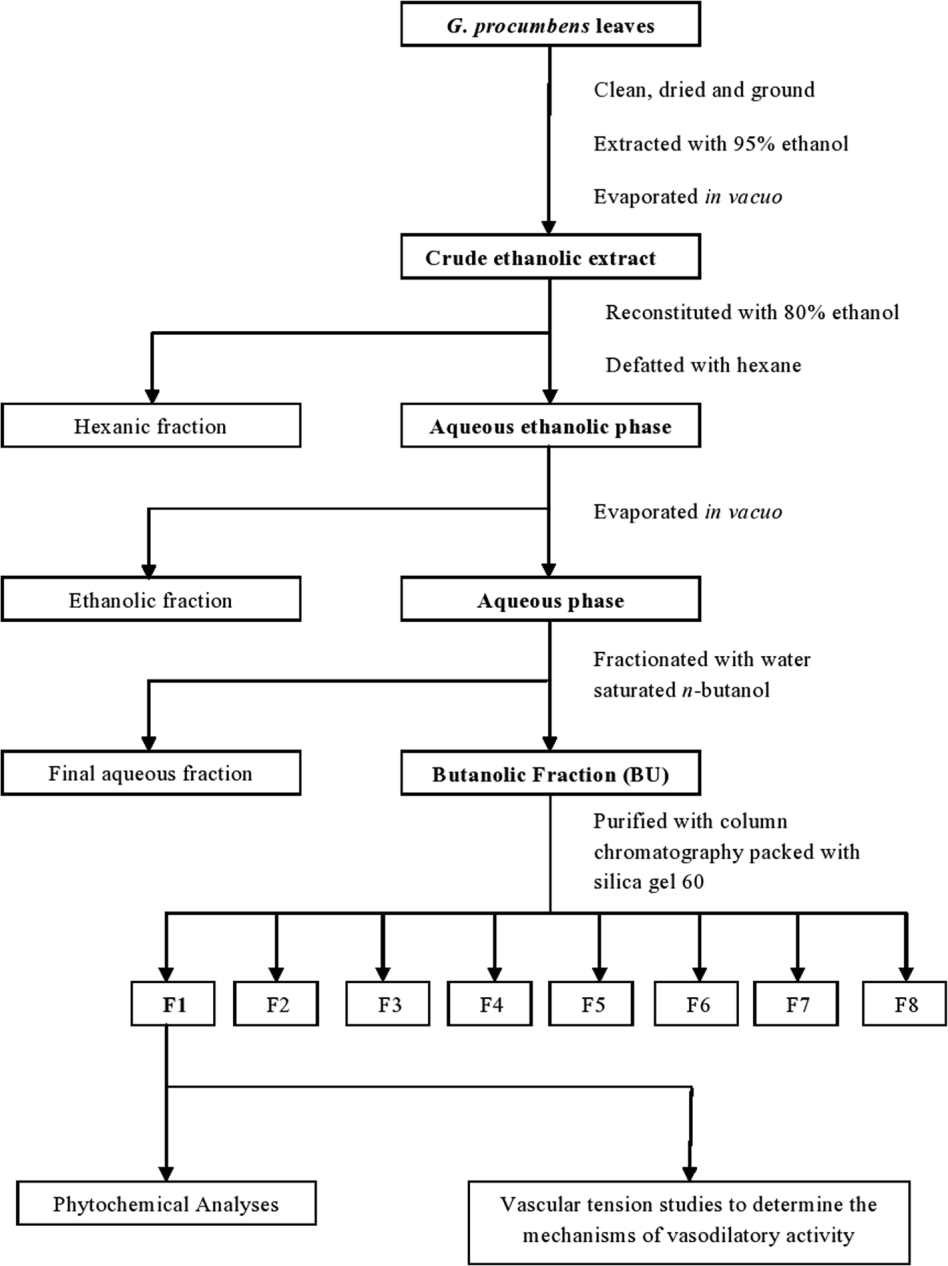
**Summary of extraction and purification of *G. procumbens *leaves.** Dried ground leaves were extracted with 95% ethanol and successively partitioned with different solvents to obtained the butanolic fraction (BU), which was then further purified into 8 sub-fractions (F1 – F8). Fraction 1 was subjected to further studies due to its highest vasodilatory activities.

The BU was further purified by using column chromatography packed with silica gel 60 (0.063 – 0.200 nm) as the stationary phase. It was dissolved in ethanol and applied to the packed column. Eight different solvent mixtures that consisted of different proportions of ethyl acetate, methanol and water were used as the mobile phases of the column chromatography with the polarities increasing from mobile phases 1 to 8. Each sub-fraction obtained was tested for vasorelaxing activities and the most active fraction was subjected to further studies. Figure 1 shows the flow chart of extraction, fractionation and purification procedures of the crude extract of *G. procumbens* leaves.

### Phytochemical screening

The sub-fraction that achieved the highest activity in the vasorelaxing effect was subjected to phytochemical screening by thin layer chromatography (TLC). The stationary phase used was TLC plate pre-coated with silica gel 60 F_254_ on aluminium sheets and the mobile phase used was a mixture of ethyl acetate (75%), methanol (10%), deionised water (10%) and formic acid (5%). Naturstoff reagent, ninhydrin reagent, and diphenylamine-aniline-phosphoric acid reagent were used for the detection of flavonoids, proteins, and sugar moieties respectively.

### *In vitro* vascular tension studies

Male SD rats weighing 250 – 300 g were sacrificed by cervical dislocation and the thoracic aorta was located. The throracic aorta was isolated and immediately placed in an oxygenated K-H solution made up of the following composition (in mM): NaCl, 118.0; KCl, 4.70; CaCl_2_, 1.25; MgSO_4_, 1.2; KH_2_PO_4_, 1.2; NaHCO_3_, 24.9 and glucose, 11.1. Adhering fat and connective tissue were carefully removed and the aorta was cut into rings of 1 – 3 mm in length. The aortic ring was mounted by 2 stainless steel holders in an organ bath containing 10 ml K-H solution and continuously bubbled with 95% oxygen and 5% carbon dioxide gas mixture. The K-H solution was maintained at 37°C by a circulating water bath system. One of the holders was served as an anchor while the other side was connected to a force transducer to measure the isometric tension developed. The force transducer was connected to a Powerlab system which displayed the responses onto the screen of a personalised computer that was installed with the software Powerlab Chart version 5.0.

A basal tension of 1 g was applied to each ring. Each preparation was allowed to equilibrate for 60 minutes with the changing of the K-H solution every 15 minutes before the start of the experiments. Aortic rings were stimulated with KCl (6 x 10^-2^ M) for 3 times to confirm the production of reproducible contractile responses. Aortic rings were denuded to remove the endothelial layer in some preparations by inserting a pair of forceps into the lumen of the aorta and gently rotating them. The viability of each aortic ring was validated by precontraction of PE (1 x 10^-6^ M) and relaxed by ACh (1 x 10^-5^ M) just before the experiment. Relaxation of ≥ 70% indicated the presence of a functional or intact endothelial layer while the lack of relaxation indicated the successful removal of the layer.

### Effects of BU and sub-fractions (F1 – F8) on aortic rings precontracted with PE or KCl

The intact aortic rings were precontracted by the addition of PE (1 x 10^-6^ M) or KCl (8 x 10^-2^ M). After the tonic responses or contractions became stable, increasing concentrations of the test fractions (10^-7^ – 10^-2^ g/ml) were added cumulatively. F1 was subjected to further studies due to its highest activity in producing the vasorelaxing effect.

### Effects of F1 on calcium (Ca^2+^) channel activities

In order to investigate whether F1 could interfere with the influx of Ca^2+^ through voltage-dependent calcium channel (VDCC), aortic rings were exposed to Ca^2+^-free solution in the presence of potassium ion (K^+^; 6 x 10^-2^ M). The preparation of the solution had the same composition with the normal K-H solution except that CaCl_2_ was excluded and EDTA (1 x 10^-4^ M) added to eliminate the extracellular Ca^2+^. Each aortic ring was exposed to this solution for 60 minutes. Two successive cumulative concentration-response curves for Ca^2+^ (1 x 10^-4^ – 1 x 10^-2^ M) were obtained. The first curve was obtained without the incubation of F1, after which the aortic ring was washed and allowed to equilibrate for 60 minutes in the solution. Two different concentrations of F1 (2.0 mg/ml and 4.0 mg/ml) were added to the organ bath and allowed to incubate for 30 minutes, after which the second curve was obtained. Each aortic ring was exposed to only one concentration of F1.

To investigate the inhibitory effect of F1 on receptor-operated calcium channel (ROCC), Ca^2+^- and K^+^-free K-H solution was used in the experiment. The composition of the solution was the same with Ca^2+^-free K-H solution except that 1.2 mM of NaH_2_PO_4_ was used to replace the 1.2 mM of KH_2_PO_4_. After the equilibration, PE (1 x 10-^6^ M) was added to induce a transient contraction. As the contraction became stable, cumulative concentrations of Ca^2+^ (1 x 10^-4^ – 1 x 10^-2^ M) were added to obtain the first curve. After that, the aortic ring was washed with normal K-H solution and incubated for 45 minutes. The solution was then replaced with Ca^2+^- and K^+^-free K-H solution. Two different concentrations of F1 (2.0 mg/ml and 4.0 mg/ml) were added to the organ bath and allowed to incubate for 30 minutes. Cumulative concentrations of Ca^2+^ were added to obtain the second curve. Each aortic ring was exposed to only one concentration of F1.

To elucidate whether the F1 could affect the release of Ca^2+^ from intracellular store, NA and caffeine were used. The aortic ring was exposed to Ca^2+^- free K-H solution for 15 minutes after the equilibration in normal K-H solution. Noradrenaline (1 x 10^-6^ M) or caffeine (4.5 x 10^-2^ M) was added to induce a transient contraction. The ring was then washed and incubated with normal K-H solution for 45 minutes to replenish the intracellular Ca^2+^ store. After that, the medium was replaced with Ca^2+^- free K-H solution and allowed to equilibrate for 15 minutes. Noradrenaline or caffeine was added after the incubation with F1 (2.0 mg/ml and 4.0 mg/ml) for 15 minutes. Each aortic ring was exposed to only one concentration of F1.

### Effects of F1 on potassium (K^+^) channel activities

In order to investigate whether the F1 could affect the K^+^ channel opening during vasorelaxation, 4-aminopyridine (1 mM), a blocker of the voltage-activated K^+^ channel; TEA (1 mM), a selective blocker to Ca^2+^-activated K^+^ channel; and glibenclamide (10 μM), a blocker of ATP-sensitive K^+^ channel were used in the experiments. All of the blockers were added in the organ bath 15 minutes before the aortic rings were precontracted with PE. After the tonic contractions had become stable, increasing concentrations of F1 (10^-7^ – 10^-2^ g/ml) were added cumulatively. The relaxation curves obtained were then compared with the control curve which were not treated with any of the blockers. Each aortic ring was exposed to only one blocker [18].

### Effects of F1 on the endothelium-denuded aortic rings precontracted with PE, NO and prostacyclin productions, and guanylyl cyclase activity

To study the involvement of endothelium in the vasorelaxation effect of F1, endothelium-denuded aortic rings were used. The denuded aortic rings were precontracted with PE before cumulative doses of F1 were added. After the tonic contractions became stable, increasing concentrations of F1 (10^-7^ – 10^-2^ g/ml) were added cumulatively.

The possible inhibitory effects of F1 on the release of two endothelium-derived releasing factor (EDRF), NO and prostacyclin, as well as its effect on the guanylyl cyclase, a key enzyme in the NO-mediated vasodilation pathway, were also investigated. In these series of experiments, the NO synthase inhibitor, L-NAME; the non-selective inhibitor of prostacyclin production, indomethacin; as well as two of the guanylyl cyclase inhibitors; ODQ and methylene blue, were used.

After the endothelium-intact aortic rings had equilibrated, the drugs were added to the organ bath 15 minutes before the addition of PE. As the PE-induced contraction reached a steady stage, increasing concentration of F1 (10^-7^ – 10^-2^ g/ml) were added cumulatively. The relaxation curves obtained were compared to the control curve [19]. Each aortic ring was exposed to only one type of drug.

### Effects of F1 on autonomic receptor activities

To study whether the vasodilation activity of F1 is related to autonomic receptors, propranolol (1 μM), a beta adrenoceptor blocker and atropine (1 μM), a muscarinic receptor blocker, were used. Both drugs were added to the organ bath 15 minutes before the addition of PE. After the tonic contractions caused by PE became stable, increasing concentrations of F1 (10^-7^ – 10^-2^ g/ml) were added cumulatively. Each aortic ring was exposed to only one type of drug.

### Statistical analysis

Changes in the relaxation were calculated as percentage change of maximal contraction induced by PE or KCl. The pIC_50_ value is calculated as the concentration of BU or F1 to produce 50% reduction of the maximal contraction induced by PE or KCl. For the cumulative dose–response curves of Ca^2+^, data from experiments in the presence or absence of the test fraction were compared. The maximal contraction (E_max_) obtained in the 1st response curve of Ca^2+^ was taken as 100% and all the following contractions were calculated as a percentage of these values. The concentration of Ca^2+^ required to produce 50% of the maximal contraction was taken as pEC_50_ (−log EC_50_).

In the experiment that involved NA and caffeine, changes in NA- or caffeine-induced contractions in the presence of F1 were expressed as percentages of the initial contraction before incubation of F1. All the analyses were performed by computer program Graphpad Prism 5 using non-linear regression of concentration-response curve. All values are expressed as mean ± S.E.M for *n* numbers of rings. Statistical differences are evaluated by Student *t*-test. A probability level of less than 0.05 (p < 0.05) is considered to be significantly different.

## Results

### Extraction and purification

Extraction of dried *G. procumbens* leaves with 95% ethanol and the subsequent fractionation lead to the production of various fractions with different percentage of yields. The yield obtained from BU was approximately 2%. Further purification of BU using column chromatography resulted in 8 sub-fractions (F1 – F8) of which F1, F2 and F3 contributed to most of the yields, accounting for 20%, 24% and 35% respectively.

### Phytochemical screening

High amount of flavonoids, which fluoresce under ultra-violet light at 365 nm after spraying with Naturstoff reagent, were detected.

### Effects of BU and sub-fractions (F1 – F8) on aortic ring precontracted with PE or KCl

**Figure 2:**
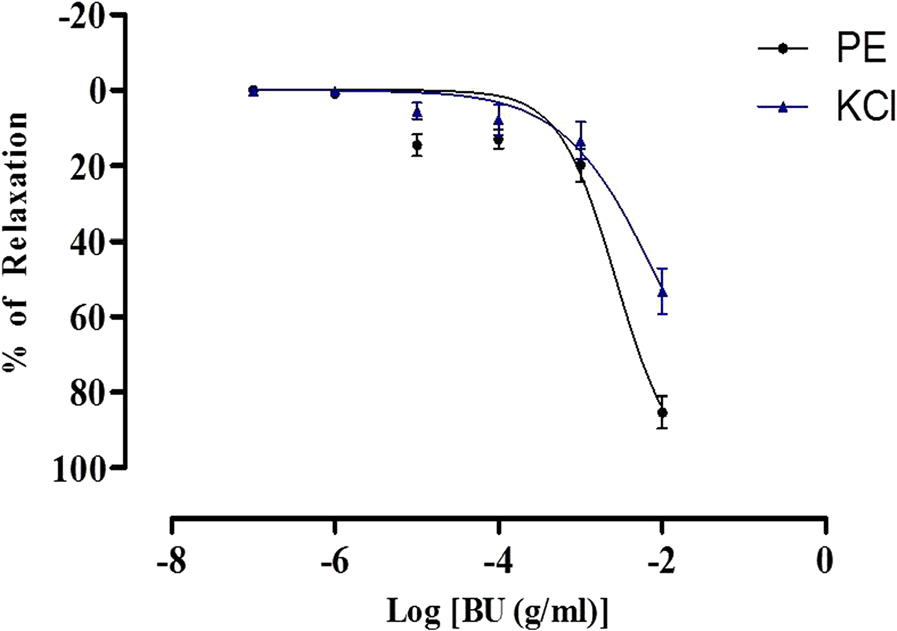
**Concentration-relaxation curves for the BU fraction in the isolated rat aortic rings.** The BU-induced relaxation was studied on endothelium-intact aortic rings precontracted with either PE or KCl. Values are mean ± S.E.M (n = 8).

The BU fraction at the concentrations ranging from 10^-7^ – 10^-2^ g/ml significantly (p < 0.05) inhibited the sustained tonic contraction induced by PE and KCl in a concentration-dependent manner. The maximal relaxation responses caused by BU, or R_max_, in the aortic rings precontracted with PE and KCl were 85.39 ± 4.26% and 53.30 ± 6.14%, respectively and the pIC_50_ values were 2.57 ± 0.06 and 2.06 ± 0.08, respectively (Figure 2).

**Figure 3:**
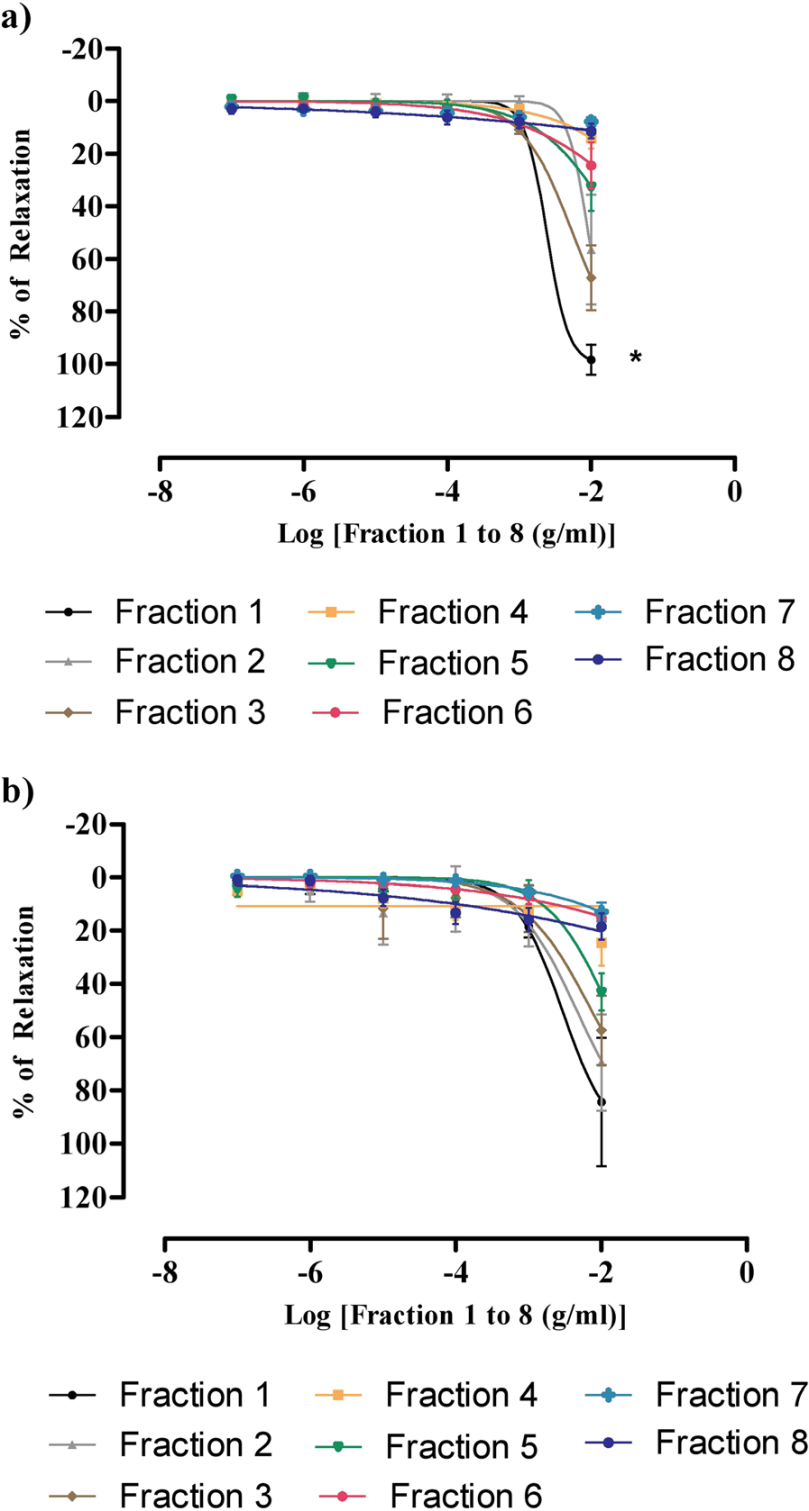
**Concentration-relaxation curves for the 8 sub-fractions in the isolated rat aortic rings.** The fractions-induced relaxations were studied on endothelium-intact aortic rings precontracted with either **(a)** PE or **(b)** KCl. Values are mean ± S.E.M (n = 6), *P < 0.05 compared with other sub-fractions.

All the sub-fractions (F1 to F8) of BU obtained from the column chromatography at concentrations ranging from 10^-7^ - 10^-2^ g/ml significantly inhibited the sustained tonic contractions induced by PE. The R_max_ value achieved by F1 was 98.39 ± 5.79% with pIC_50_ value of 2.62 ± 0.002 and both of these values were significantly different (p < 0.05) with that shown by all other sub-fractions (Figure 3, Table 1)

**Table 1:** The effects of BU-derived sub-fractions in aortic rings precontracted with PE or KCl

**Fraction**	**PE**		**KCl**	
	**R**_**max **_**(%)**	**pIC**_**50**_	**R**_**max **_**(%)**	**pIC**_**50**_
1	98.39 ± 5.79*	2.62 ± 0.09*	84.40 ± 24.04	2.54 ± 0.15
2	56.57 ± 20.90	2.04 ± 0.35	69.54 ± 18.08	2.34 ± 0.18
3	67.21 ± 12.33	2.24 ± 0.08	57.45 ± 13.09	2.13 ± 0.15
4	14.02 ± 3.92	-	24.54 ± 8.67	-
5	32.47 ± 9.12	-	43.01 ± 6.93	-
6	24.35 ± 8.74	-	15.15 ± 3.72	-
7	7.84 ± 1.57	-	12.85 ± 3.52	-
8	11.38 ± 2.75	-	18.38 ± 4.99	-

Values are means ± S.E.M (n = 6). *P < 0.05 compared with other sub-fractions.

For the aortic ring precontracted with KCl, F1 also achieved the highest percentage of relaxation with the R_max_ = 84.40 ± 24.04% and pIC_50_ = 2.54 ± 0.003, respectively. There was no significant difference in the percentage of relaxation that caused by F1 if compared with F2 and F3. (Figure 3, Table 1).

In view of the finding that the relaxant effect of F1 is the highest and significantly different from that of the BU and other fractions, it was subjected to further studies.

### Effects of F1 on calcium channel activities

**Figure 4:**
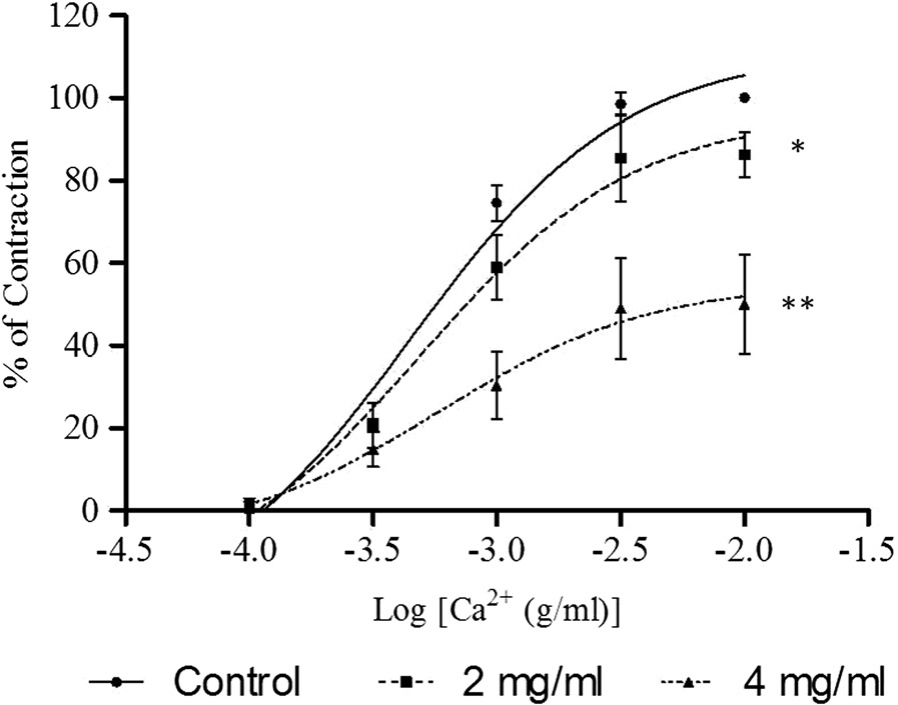
**Effect of F1 on CaCl**_**2**_**-induced contraction in Ca**^**2+**^**-free solution containing high K**^**+ **^**(60 mM).** Concentration-response curves for CaCl_2_ were determined in aortic rings in the absence and presence of F1 (2 or 4 mg/ml). Values are mean ± S.E.M. (n = 6). *P < 0.05 and **P < 0.01 compared with control (without F1).

As shown in Figure 4, CaCl_2_ caused a concentration-dependent contraction of aortic rings that had been exposed to a Ca^2+^-free medium with high concentration of K^+^. The E_max_ values were significantly reduced by preincubation of the rings with F1 at 2.0 x 10^-3^ mg/ml (p < 0.05) and 4.0 x 10^-3^ mg/ml (p < 0.01) in a concentration- dependent manner (Table 2). The pEC_50_ values were also reduced in the presence of F1.

**Table 2:** **The effects of F1 on E**_**max **_**and pEC**_**50 **_**values for CaCl**_**2**_

**F1 (mg/ml)**	**Ca**^**2+**^**-free solution containing high K**^**+**^		**Ca**^**2+ **^**and K**^**+**^**-free solution containing PE**	
	**E**_**max **_**(%)**	**pEC**_**50**_	**E**_**max **_**(%)**	**pEC**_**50**_
0	100	3.35 ± 0.08	100	3.73 ± 0.21
2.0	86.33 ± 5.44*	3.31 ± 0.19	75.33 ± 10.33**	3.61 ± 0.27
4.0	50.04 ± 12.06**	3.24 ± 0.42	44.93 ± 11.46**	3.34 ± 0.48

Values are means ± S.E.M (n = 6). *P < 0.05 and **P < 0.01 compared with control (0 mg/ml of F1.

**Figure 5:**
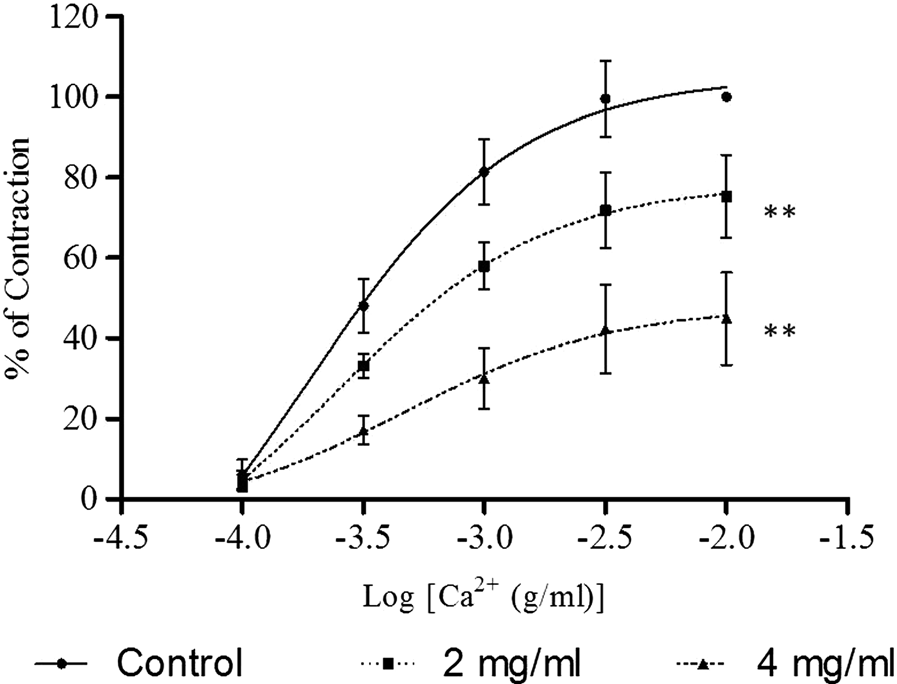
**Effect of F1 on CaCl**_**2**_**-induced contraction in Ca**^**2+**^**- and K**^**+**^**-free solution containing PE.** Concentration-response curves for CaCl_2_ were determined in aortic rings in the absence and presence of F1 (2 or 4 mg/ml). Values are mean ± S.E.M. (n = 6). **P < 0.01 compared with control (without F1).

**Figure 6:**
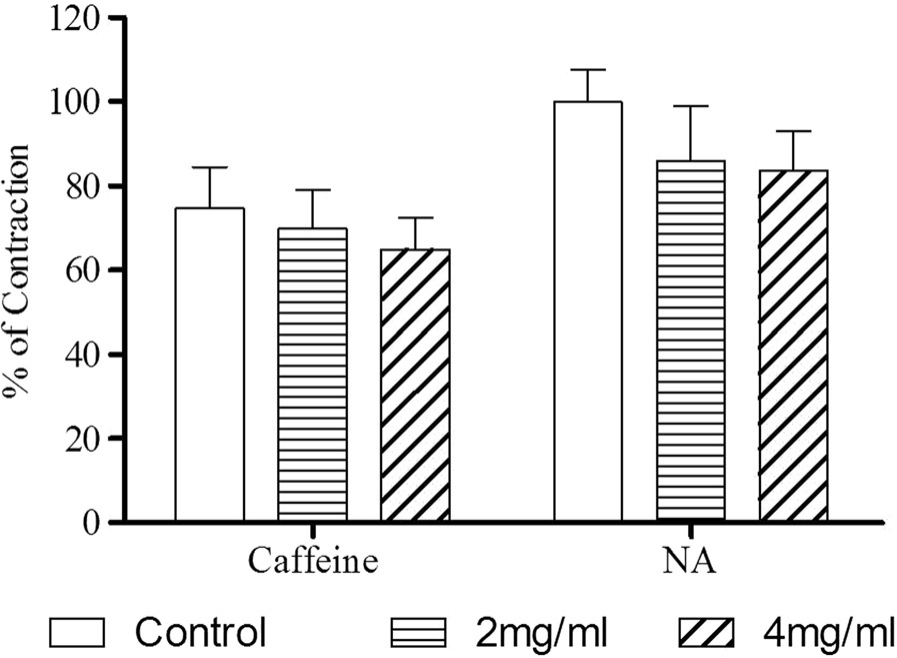
**Effect of F1 on NA- and caffeine-induced contraction in Ca**^**2+**^**-free solution.** Aortic rings were preincubated with vehicle (control) or BU (2 or 4 mg/ml) and NA (10^-6^ M) or caffeine (45 mM) was added to trigger the contractions. Values are mean ± S.E.M. (n = 6).

In a Ca^2+^- and K^+^-free medium, Ca^2+^ (1 x 10^-4^ – 1 x 10^-2^ M) was cumulatively added to the organ bath after PE had induced a stable tonic contraction. Sustained contraction was generated which increased with the concentration of Ca^2+^. The Ca^2+^- induced contraction was significantly inhibited by the preincubation of the aortic ring with F1 (2.0 x 10^-3^ and 4.0 x 10^-3^ mg/ml) in a concentration-dependent manner (Figure 5). F1 significantly (p < 0.01) suppressed the E_max_ values of CaCl_2_ with the reduction of the pEC_50_ values (Table 2). Results in Figure 6 show that the contractions induced by either NA or caffeine in Ca^2+^-free solution were not significantly affected by F1.

### Effects of F1 on potassium channel activities

**Figure 7:**
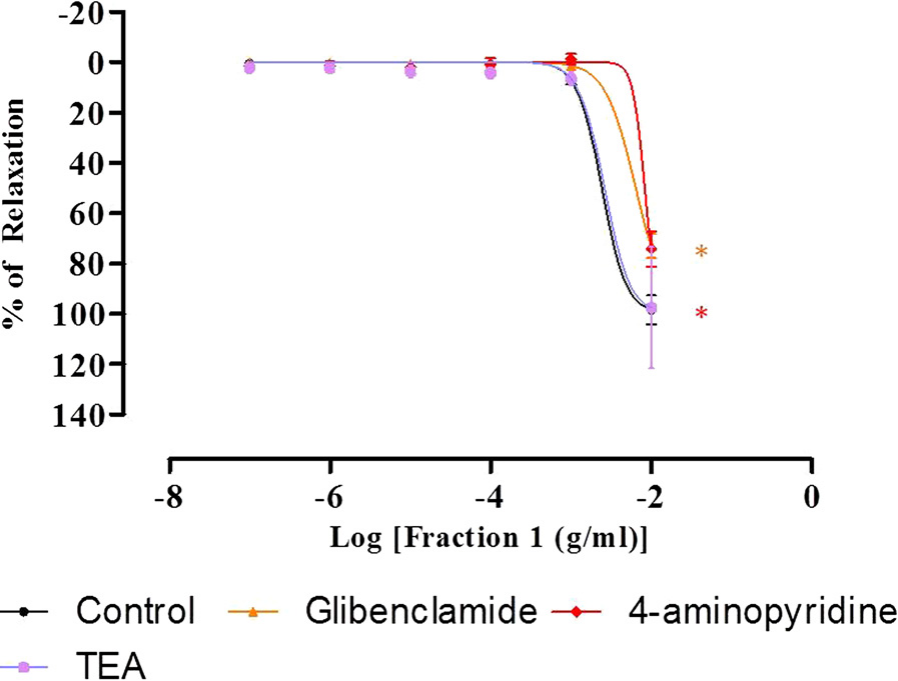
**Concentration-relaxation curves for F1 in the isolated rat aortic rings.** The F1-induced relaxation was studied on PE-precontracted endothelium-intact aortic ring preincubated with TEA or glibenclamide or 4-aminopyridine. Values are mean ± S.E.M (n = 6). *P < 0.05 compared with control (without blocker).

As shown in Figure 7, preincubation with glibenclamide and 4-aminopyridine caused significant reductions in the R_max_ values caused by F1. Similarly, the pIC_50_ values were reduced in the presence of these 2 drugs (Table 3). However, there was no significant difference found between the F1-induced relaxation curves of the aortic rings preincubated with TEA and the control curve. The R_max_ and pIC_50_ values were also not significantly altered by the incubation with TEA.

**Table 3:** **The R**_**max **_**and pIC**_**50 **_**values of F1 in aortic rings pretreated with various drugs**

**Drugs**	**R**_**max **_**(%)**	**pIC**_**50**_
Control	98.39 ± 5.79	2.62 ± 0.09
TEA	97.61 ± 24.04	2.58 ± 0.30
Glibenclamide	73.23 ± 4.84*	2.19 ± 0.06*
4-aminopyridine	74.31 ± 6.89*	2.08 ± 0.05*
L-NAME	82.77 ± 13.12	2.49 ± 0.12
Indomethacin	68.15 ± 5.96*	2.37 ± 0.05*
ODQ	88.46 ± 6.49	2.51 ± 0.14
Methylene blue	107.98 ± 9.29	2.85 ± 0.17
Atropine	92.12 ± 2.64	2.53 ± 0.07
Propanolol	93.02 ± 8.93	2.46 ± 0.09

Values are means ± S.E.M (n = 6), *P < 0.05 compared with control (without any drugs).

### Effects of F1 on the endothelium-denuded aortic rings precontracted with PE, NO and prostacyclin productions, and guanylyl cyclase activity

**Figure 8:**
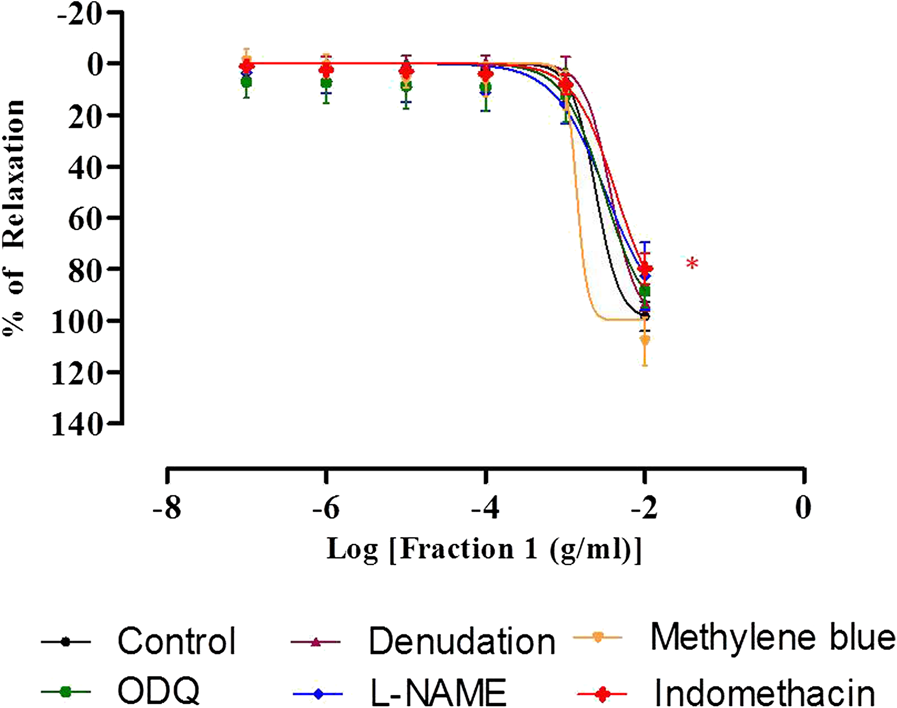
**Concentration-relaxation curves for F1 in the isolated rat aortic rings.** The F1-induced relaxation was studied on endothelium-intact, endothelium-denuded aortic rings and endothelium-intact aortic rings preincubated with L-NAME or ODQ or methylene blue or indomethacin. Values are mean ± S.E.M (n = 6).

The R_max_ and pIC_50_ of endothelium-denuded aortic rings are 93.28 ± 3.02% and 2.44 ± 0.09 respectively, which are not significantly different from the control values of 98.39 ± 5.79% and 2.62 ± 0.09 respectively. Besides that, the vasorelaxing effect (R_max_ and pIC_50_) induced by F1 were also not significantly affected by preincubation of L-NAME, ODQ, and methylene blue. Nonetheless, preincubation of indomethacin caused significant reductions in both the R_max_ and pIC_50_ values of F1 (Figure 8, Table 3).

### Effects of F1 on the autonomic receptor activities

**Figure 9:**
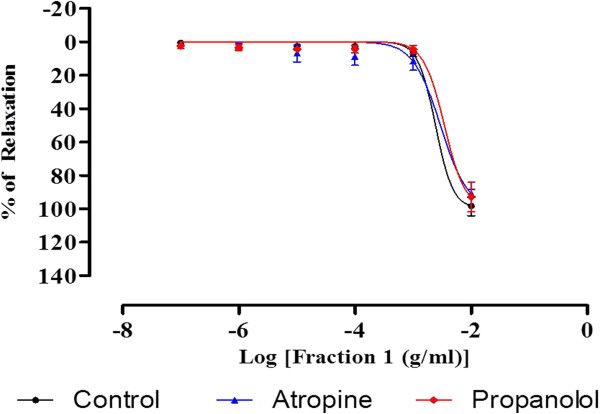
**Concentration-relaxation curves for F1 in the isolated rat aortic rings.** The F1-induced relaxation was studied on PE-precontracted endothelium-intact aortic rings preincubated with atropine or propanolol. Value are mean ± S.E.M (n = 6).

As shown in Figure 9, the vasorelaxing effects of F1 against PE-precontracted aortic rings were not significantly inhibited by the preincubation with atropine or propranolol. In both experiments, the R_max_ and pEC_50_ values did not show any significant difference with that of the control (Table 3).

## Discussion

In the present study, BU was found to cause relaxation on the endothelium-intact aortic rings that had been precontracted with PE or KCl (Figure 1). This confirms the results reported in a previous study [17] that BU may reduce BP by vasodilation. BU was then further purified by column chromatography using solvent mixtures of different polarities. It was found that F1 demonstrated the highest vasodilatory activity and the effect was more profound (p < 0.05) when compared with BU-induced vasorelaxation. This indicates that most of the putative active compounds of BU appear to be concentrated in F1. Phenylephrine activates the α_1_-adrenoceptors in the VSM to cause the muscle contraction involving phospholipase C (PLC) and 1,2-diacylglycerol (DAG) [20,21] while KCl causes the depolarisation of the VSM cell membrane, opening of VDCC and influx of Ca^2+^ in order to cause contraction [22]. Both BU- and F1 induced relaxation in aortic rings precontracted with PE or KCl, suggesting that they might inhibit both the activation of α_1_-adrenoceptors and opening of the VDCC.

It is well known that vasoconstriction is initiated by elevated levels of the free cytoplasmic Ca^2+^ which can either be due to the influx of extracellular Ca^2+^ upon opening of the VDCC and ROCC located on the cell membrane [22,23] or the release of Ca^2+^ from sarcoplasmic reticulum (SR) [24,25]. To investigate whether F1 could interfere with the Ca^2+^ influx through VDCC, CaCl_2_-induced contraction in Ca^2+^-free with high K^+^ medium was used. Under high concentrations of K^+^, the added Ca^2+^ will cause the influx of Ca^2+^ through the VDCC [22]. Whereas to investigate whether F1 could interfere with the Ca^2+^ through ROCC, Ca^2+^- and K^+^-free medium containing PE was used. Addition of PE in the Ca^2+^-and K^+^-free medium will cause the release of Ca^2+^ from intracellular store and the entry of Ca^2+^ into the cells through ROCC since VDCC are inactivated in the absence of K^+^[26,27]. Results in Figures 3 and 4 show that preincubation with F1 significantly decreased the E_max_ of the CaCl_2_-induced contractions in both media, suggesting that the vasodilatory effect of F1 is caused by blocking the influx of Ca^2+^ via VDCC and ROCC.

Caffeine and NA were used to study the release of Ca^2+^ from intracellular sarcoreticular stores. Caffeine can stimulate the ryanodine receptors [28] and NA can trigger the activation of IP_3_[29] receptor located at the SR in order to cause the release of the Ca^2+^ from SR. In the present study, it is found that the vasoconstriction induced by both caffeine and NA were not affected by F1 (Figure 5). Therefore, the vasodilatory effect of F1 does not seem to be related to inhibition of ryanodine or IP_3_ receptors.

Potassium channels play a very crucial role in controlling the vascular contractility. Efflux of K^+^ due to the opening of the K^+^ channels in VSM will bring about membrane hyperpolarisation, which in turn, will lead to the closure of the VDCC and reduction of the Ca^2+^ entry, and finally cause vasodilation [30]. In contrast, closure of the K^+^ channels will cause membrane depolarisation and vasoconstriction [31]. Four distinct types of K^+^ channels have been identified in VSM, namely voltage-activated K^+^ channel, Ca^2+^-activated K^+^ channels, ATP-sensitive K^+^ channels, and inward rectifier K + channels) [32]. As demonstrated by the results in Figure 6, F1 may be able to enhance or stimulate the opening of both voltage-activated and ATP-sensitive K^+^ channels, since preincubation with glibenclamide and 4-aminopyridine significantly attenuated its vasodilatory effect. On the other hand, Ca^2+^-activated K^+^ channel may not be involved in the vasodilatory effect of F1 since R_max_ value of F1 was not significantly altered with the preincubation of TEA.

Nitric oxide is the primary EDRF formed in the endothelium that diffuses to the VSM to activate the soluble guanylyl cyclase. This enzyme will then catalyse the formation of cyclic guanosine monophosphate (cGMP), which in turn activates the protein kinase G (PKG). The PKG will cause the phophorylation of myosin light-chain kinase (MLCK) and subsequently decrease its activity, leading to the dephosphorylation of myosin light chain and thus causing vasorelaxation [33]. In addition, NO also participates in the regulation of the level of smooth muscle free Ca^2+^, which is the primary determinant of contractions [34]. Prostacyclin is the lipid compound derived enzymatically from fatty acid and it is the first EDRF that was discovered earlier than NO [35]. It is synthesised in both VSM and endothelial cells [36]. Results from the present studies show that removal of endothelium did not cause significant changes in the R_max_ and pIC_50_ of F1-induced relaxation. This indicates that endothelium may not be involved in the vasodilatory effect of F1. Similarly, there were also no significant changes in the R_max_ and pIC_50_ values for the aortic rings preincubated with L-NAME, ODQ, and methylene blue (Figure 7). These findings imply that F1 does not affect the endothelium-derived NO production as well as the NO-mediated pathway. To the contrary, significant reductions in both R_max_ and pIC_50_ were observed in the aortic ring preincubated with indomethacin, the inhibitor of the cyclo-oxygenase that participates in the prostacyclin synthesis. Prostacyclin is synthesised in both endothelium and VSM. The vasodilatory effect of F1 was attenuated by the inhibition of cyclo-oxygenase but not by the removal of endothelium, suggesting that F1 may stimulate the release of prostacyclin from VSM.

The beta adrenoceptor acts by coupling with G protein. The activation of beta adrenoceptor stimulates adenylyl cyclase which functions as a catalyst for the conversion of adenosine triphosphate to cyclic adenosine monophosphate. This in turn leads to the activation of protein kinase A and vasodilation [37]. Muscarinic receptors are stimulated by the postganglionic cholinergic neurons of either the parasympathetic or the sympathetic cholinergic systems that mediate smooth muscle relaxation, glandular secretion, and modulation of cardiac rate and force [38]. Results from the present study show that there were no significant changes in the R_max_ and pEC_50_ in the aortic rings preincubated with propanolol and atropine (Figure 8). This suggests that beta adrenoceptor and muscarinic receptor may not be involved in the vasodilatory effect of F1.

Phytochemcial screening of the compounds present in F1 detected high amount of flavonoids. Flavonoids are polyphenolic compounds that occur naturally in vegetables, fruits, seeds, nuts, barks and are an integral part of the human diet [39]. Flavonoids belong to the low molecular weight group of polyphenol substances. The biochemical activities of flavonoids and their metabolites depend on their chemical structure and relative orientation of various moieties on the molecule. They can be grouped into 8 major classes by their chemical structures that include flavones, flavanones, flavonols, catechins, anthocyanidins, isoflavones, dihydroflavonols, and chalcones [40]. Depending on their chemical structures, these flavonoids have been found to possess a wide range of pharmacological activities, such as antihypertensive [41,42] and vasodilatory [43,44] activities.

In the present study, although the phytochemical screening technique did not reveal the actual chemical structures of the active compounds in F1, it helped to show that the majority of compounds present in F1 are flavonoids. Based on the evidence that F1 exhibits vasodilatory activity and as well as containing flavonoids, it is logical to postulate that the vasodilatory effect of F1 could possibly be attributed to the presence of flavonoid. However more experiments need to be carried out in order to confirm this association and to identify the structure of the active compounds.

## Conclusions

This study demonstrates conclusively that leaves of *G. procumbens* contain potent vasodilatory substances, more of them being found in the F1 subfraction of the BU fraction. These results also further confirm previous findings that the vasodilatory effects of BU were brought about by blocking the calcium channels. In addition to the Ca^2+^ channel-blocking activities, the active compounds were also found to be able to open K^+^ channel as well as to stimulate the production of prostacyclin. Phytochemical analyses reveal that the putative compounds are probably flavonoids in nature.

## Competing interests

The authors declare that they have no competing interests.

## Authors’ contributions

NHK participated in the design of study and carried out all the extraction, purification, isolated aortic rings experiments and drafted the manuscript. PTF carried out the extraction process. LSK and HSZ participated in the design of study and edited the manuscript. All authors have read and approved the final manuscript.

## Pre-publication history

The pre-publication history for this paper can be accessed here:

http://www.biomedcentral.com/1472-6882/13/188/prepub
